# (*E*,*E*)-6,6′-Dimeth­oxy-2,2′-[*o*-phenyl­ene­bis(nitrilo­methyl­idyne)]diphenol

**DOI:** 10.1107/S1600536809008757

**Published:** 2009-03-14

**Authors:** Yong Wang, Hong-Gang Li, Handong Yin, Guo-Dong Wei, Xiao Wang

**Affiliations:** a Department of Chemistry, Liaocheng University, Liaocheng 252059, People’s Republic of China; b Clinical Medicine Department, Weifang Medical University, Shangdong 261042, People’s Republic of China; c Shandong Donge Experimental High School, Shandong 252200, People’s Republic of China

## Abstract

In the title compound, C_22_H_20_N_2_O_4_, the central benzene ring forms dihedral angles of 3.2 (2) and 61.1 (1)° with the two outer substituted benzene rings. Intra­molecular O—H⋯N hydrogen bonds are formed by both hydroxyl groups.

## Related literature

For background literature concerning salen-type ligands, see: Zhang *et al.* (1990 ▶). For related structures, see: Lo *et al.* (2006 ▶).
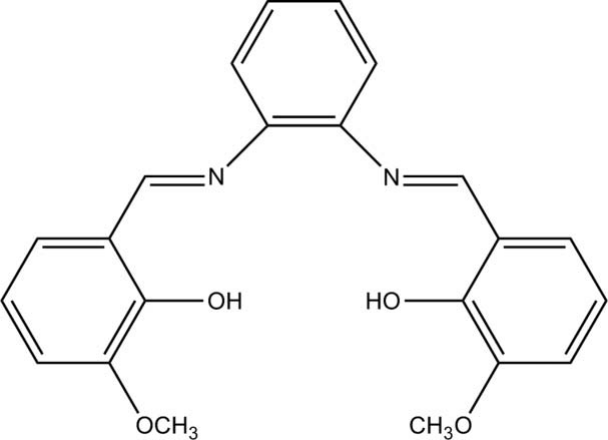


## Experimental

### 

#### Crystal data

C_22_H_20_N_2_O_4_
*M* *_r_* = 376.40
Monoclinic, 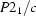

*a* = 6.5863 (8) Å
*b* = 16.726 (2) Å
*c* = 17.023 (3) Å
β = 97.926 (2)°
*V* = 1857.3 (4) Å^3^
*Z* = 4
Mo *K*α radiation
μ = 0.09 mm^−1^
*T* = 298 K
0.33 × 0.15 × 0.09 mm

#### Data collection

Siemens SMART CCD diffractometer
Absorption correction: multi-scan (*SADABS*; Sheldrick, 1996 ▶) *T*_min_ = 0.970, *T*_max_ = 0.992
9269 measured reflections
3263 independent reflections
1217 reflections with *I* > 2σ(*I*)
*R*_int_ = 0.098

#### Refinement

*R*[*F* ^2^ > 2σ(*F* ^2^)] = 0.050
*wR*(*F* ^2^) = 0.128
*S* = 0.82
3263 reflections
257 parameters
H-atom parameters constrained
Δρ_max_ = 0.15 e Å^−3^
Δρ_min_ = −0.16 e Å^−3^

### 

Data collection: *SMART* (Siemens, 1996 ▶); cell refinement: *SAINT* (Siemens, 1996 ▶); data reduction: *SAINT*; program(s) used to solve structure: *SHELXS97* (Sheldrick, 2008 ▶); program(s) used to refine structure: *SHELXL97* (Sheldrick, 2008 ▶); molecular graphics: *SHELXTL* (Sheldrick, 2008 ▶); software used to prepare material for publication: *SHELXTL*.

## Supplementary Material

Crystal structure: contains datablocks I, global. DOI: 10.1107/S1600536809008757/bi2352sup1.cif

Structure factors: contains datablocks I. DOI: 10.1107/S1600536809008757/bi2352Isup2.hkl

Additional supplementary materials:  crystallographic information; 3D view; checkCIF report

## Figures and Tables

**Table 1:** Hydrogen-bond geometry (Å, °)

*D*—H⋯*A*	*D*—H	H⋯*A*	*D*⋯*A*	*D*—H⋯*A*
O1—H1⋯N1	0.82	1.88	2.605 (4)	146
O3—H3⋯N2	0.82	1.82	2.542 (3)	146

**Fig. 1.**
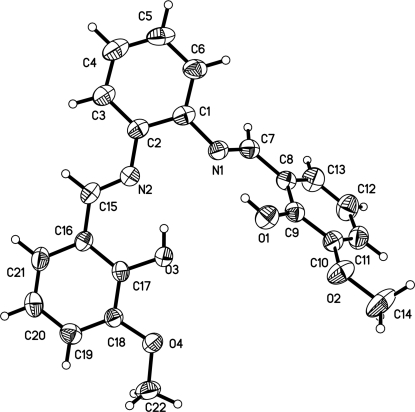
Molecular structure of the title compound showing 30% probability displacement ellipsoids for non-H atoms.

## supplementary crystallographic information

### Comment

Salen-type ligands are amongst the oldest ligands in coordination chemistry and have received considerable interest since Jacobsen and Katsuki first reported their significant success using chiral manganese(III) salen Schiff-base catalysts in the asymmetric epoxidation of unfunctionalized olefins (Zhang, *et al.*, 1990).

The title compound (Fig. 1) was obtained by reaction of *o*-phenylenediamine and 2-hydroxy-3-methoxybenzaldehyde. The bond lengths and angles are comparable to those in the related compound *N*,*N*'-bis(3- methoxysalicylidene)phenylene-1,2-diamine (Lo *et al.*, 2006). The central benzene ring is almost coplanar with the benzene ring C16–C21; the dihedral angle between the two planes is 3.21 (22) °. However, the dihedral angle of the central benzene ring and the benzene ring C8–C13 is 61.13 (11)°. Intramolecular O—H···N hydrogen bonds are formed by both hydroxyl groups (Table 1).

### Experimental

To a solution of *o*-phenylenediamine (3 mmol) in ethanol (30 ml) was added 2-hydroxy-3-methoxybenzaldehyde (6 mmol). The mixture was refluxed with stirring for 20 min and an orange precipitate was obtained. Orange crystals suitable for X-ray diffraction analysis formed after several weeks on slow evaporation of an ethanol solution at room temperature. Elemental analysis: calculated for C_22_H_20_N_2_O_4_: C 70.20, H 5.36, N 7.44%; found: C 70.28, H 5.32, N 7.49%.

### Refinement

All H atoms were positioned geometrically and refined using a riding model with C—H = 0.93 or 0.96 Å and *U*_iso_(H) = 1.2 or 1.5*U*_eq_(C). The H atoms of the hydroxyl groups were placed in idealized positions and constrained to ride on their parent atoms with O—H = 0.82 Å and *U*_iso_(H) = 1.5*U*_eq_(O).

### Crystal data

**Table:**

C_22_H_20_N_2_O_4_	*F*(000) = 792
*M**_r_* = 376.40	*D*_x_ = 1.346 Mg m^−^^3^
Monoclinic, *P*2_1_/*c*	Mo *K*α radiation, λ = 0.71073 Å
Hall symbol: -P 2ybc	Cell parameters from 802 reflections
*a* = 6.5863 (8) Å	θ = 2.4–25.3°
*b* = 16.726 (2) Å	µ = 0.09 mm^−^^1^
*c* = 17.023 (3) Å	*T* = 298 K
β = 97.926 (2)°	Block, orange
*V* = 1857.3 (4) Å^3^	0.33 × 0.15 × 0.09 mm
*Z* = 4	

### Data collection

**Table:**

Siemens SMART CCD diffractometer	3263 independent reflections
Radiation source: fine-focus sealed tube	1217 reflections with *I* > 2σ(*I*)
graphite	*R*_int_ = 0.098
ω scans	θ_max_ = 25.0°, θ_min_ = 1.7°
Absorption correction: multi-scan (*SADABS*; Sheldrick, 1996)	*h* = −7→7
*T*_min_ = 0.970, *T*_max_ = 0.992	*k* = −19→19
9269 measured reflections	*l* = −10→20

### Refinement

**Table:**

Refinement on *F*^2^	Primary atom site location: structure-invariant direct methods
Least-squares matrix: full	Secondary atom site location: difference Fourier map
*R*[*F*^2^ > 2σ(*F*^2^)] = 0.050	Hydrogen site location: inferred from neighbouring sites
*wR*(*F*^2^) = 0.128	H-atom parameters constrained
*S* = 0.82	*w* = 1/[σ^2^(*F*_o_^2^) + (0.0396*P*)^2^] where *P* = (*F*_o_^2^ + 2*F*_c_^2^)/3
3263 reflections	(Δ/σ)_max_ < 0.001
257 parameters	Δρ_max_ = 0.15 e Å^−^^3^
0 restraints	Δρ_min_ = −0.16 e Å^−^^3^

### Special details

**Table:**

Geometry. All e.s.d.'s (except the e.s.d. in the dihedral angle between two l.s. planes) are estimated using the full covariance matrix. The cell e.s.d.'s are taken into account individually in the estimation of e.s.d.'s in distances, angles and torsion angles; correlations between e.s.d.'s in cell parameters are only used when they are defined by crystal symmetry. An approximate (isotropic) treatment of cell e.s.d.'s is used for estimating e.s.d.'s involving l.s. planes.
Refinement. Refinement of *F*^2^ against ALL reflections. The weighted *R*-factor *wR* and goodness of fit *S* are based on *F*^2^, conventional *R*-factors *R* are based on *F*, with *F* set to zero for negative *F*^2^. The threshold expression of *F*^2^ > σ(*F*^2^) is used only for calculating *R*-factors(gt) *etc*. and is not relevant to the choice of reflections for refinement. *R*-factors based on *F*^2^ are statistically about twice as large as those based on *F*, and *R*- factors based on ALL data will be even larger.

### Fractional atomic coordinates and isotropic or equivalent isotropic displacement parameters (Å^2^)

**Table:**

	*x*	*y*	*z*	*U*_iso_*/*U*_eq_	
N1	−0.1935 (4)	0.21173 (18)	0.39296 (16)	0.0568 (8)	
N2	0.0295 (4)	0.26464 (15)	0.52584 (18)	0.0579 (8)	
O1	0.0593 (4)	0.15813 (15)	0.30030 (15)	0.0719 (7)	
H1	0.0140	0.1899	0.3303	0.108*	
O2	0.1592 (4)	0.04788 (15)	0.20699 (16)	0.0868 (9)	
O3	0.2802 (3)	0.15729 (14)	0.49515 (14)	0.0676 (7)	
H3	0.1719	0.1822	0.4903	0.101*	
O4	0.6184 (3)	0.08108 (14)	0.53290 (13)	0.0698 (7)	
C1	−0.2767 (6)	0.2731 (2)	0.4365 (2)	0.0575 (10)	
C2	−0.1578 (6)	0.3022 (2)	0.5035 (2)	0.0572 (10)	
C3	−0.2322 (6)	0.3642 (2)	0.5450 (2)	0.0715 (11)	
H3A	−0.1540	0.3843	0.5903	0.086*	
C4	−0.4199 (7)	0.3955 (2)	0.5195 (3)	0.0834 (13)	
H4	−0.4689	0.4374	0.5475	0.100*	
C5	−0.5373 (6)	0.3668 (3)	0.4537 (3)	0.0797 (13)	
H5	−0.6659	0.3887	0.4371	0.096*	
C6	−0.4654 (6)	0.3057 (2)	0.4120 (2)	0.0719 (11)	
H6	−0.5451	0.2862	0.3667	0.086*	
C7	−0.3021 (5)	0.1499 (2)	0.37462 (19)	0.0581 (10)	
H7	−0.4285	0.1452	0.3928	0.070*	
C8	−0.2348 (5)	0.0871 (2)	0.3265 (2)	0.0555 (9)	
C9	−0.0623 (6)	0.0944 (2)	0.2904 (2)	0.0552 (9)	
C10	−0.0088 (6)	0.0337 (2)	0.2413 (2)	0.0590 (10)	
C11	−0.1242 (7)	−0.0340 (2)	0.2324 (2)	0.0754 (12)	
H11	−0.0887	−0.0749	0.1998	0.090*	
C12	−0.2921 (7)	−0.0425 (3)	0.2709 (3)	0.0910 (14)	
H12	−0.3674	−0.0897	0.2656	0.109*	
C13	−0.3491 (6)	0.0175 (3)	0.3167 (2)	0.0819 (12)	
H13	−0.4654	0.0119	0.3416	0.098*	
C14	0.2181 (7)	−0.0106 (2)	0.1550 (3)	0.1190 (17)	
H14A	0.2234	−0.0619	0.1805	0.178*	
H14B	0.3510	0.0023	0.1414	0.178*	
H14C	0.1202	−0.0121	0.1077	0.178*	
C15	0.1427 (6)	0.27815 (19)	0.5911 (2)	0.0595 (10)	
H15	0.1003	0.3152	0.6262	0.071*	
C16	0.3323 (5)	0.23818 (19)	0.6117 (2)	0.0522 (9)	
C17	0.3958 (5)	0.1790 (2)	0.5627 (2)	0.0516 (9)	
C18	0.5797 (5)	0.1401 (2)	0.5837 (2)	0.0543 (9)	
C19	0.7041 (5)	0.1621 (2)	0.6500 (2)	0.0668 (11)	
H19	0.8303	0.1371	0.6631	0.080*	
C20	0.6450 (7)	0.2217 (2)	0.6987 (2)	0.0743 (12)	
H20	0.7314	0.2364	0.7443	0.089*	
C21	0.4615 (6)	0.2584 (2)	0.6800 (2)	0.0709 (11)	
H21	0.4218	0.2977	0.7134	0.085*	
C22	0.8046 (5)	0.0386 (2)	0.5504 (2)	0.0837 (13)	
H22A	0.9176	0.0751	0.5522	0.125*	
H22B	0.8139	−0.0008	0.5100	0.125*	
H22C	0.8088	0.0126	0.6009	0.125*	

### Atomic displacement parameters (Å^2^)

**Table:**

	*U*^11^	*U*^22^	*U*^33^	*U*^12^	*U*^13^	*U*^23^
N1	0.064 (2)	0.059 (2)	0.0483 (18)	0.0109 (17)	0.0112 (15)	0.0045 (17)
N2	0.059 (2)	0.0576 (19)	0.057 (2)	0.0020 (16)	0.0072 (16)	−0.0030 (17)
O1	0.0748 (18)	0.0689 (18)	0.076 (2)	−0.0043 (14)	0.0235 (13)	−0.0008 (15)
O2	0.096 (2)	0.085 (2)	0.089 (2)	0.0161 (16)	0.0462 (17)	0.0009 (17)
O3	0.0697 (18)	0.0699 (17)	0.0598 (16)	0.0129 (13)	−0.0036 (13)	−0.0172 (14)
O4	0.0727 (18)	0.0755 (17)	0.0601 (16)	0.0191 (14)	0.0055 (13)	−0.0091 (15)
C1	0.067 (3)	0.054 (2)	0.054 (3)	0.009 (2)	0.020 (2)	0.016 (2)
C2	0.066 (3)	0.050 (2)	0.059 (3)	0.005 (2)	0.019 (2)	0.006 (2)
C3	0.076 (3)	0.063 (3)	0.077 (3)	0.012 (2)	0.017 (2)	−0.005 (2)
C4	0.106 (4)	0.069 (3)	0.081 (3)	0.029 (3)	0.035 (3)	0.006 (3)
C5	0.081 (3)	0.079 (3)	0.083 (3)	0.035 (3)	0.027 (3)	0.025 (3)
C6	0.077 (3)	0.080 (3)	0.059 (3)	0.024 (2)	0.013 (2)	0.015 (2)
C7	0.060 (2)	0.068 (3)	0.047 (2)	0.007 (2)	0.0104 (18)	0.010 (2)
C8	0.054 (2)	0.062 (3)	0.052 (2)	0.001 (2)	0.0114 (19)	0.008 (2)
C9	0.062 (3)	0.052 (2)	0.050 (2)	0.002 (2)	0.0035 (19)	0.007 (2)
C10	0.072 (3)	0.061 (3)	0.045 (2)	0.009 (2)	0.013 (2)	0.011 (2)
C11	0.094 (3)	0.071 (3)	0.060 (3)	0.007 (3)	0.007 (2)	−0.004 (2)
C12	0.103 (4)	0.076 (3)	0.096 (4)	−0.021 (3)	0.021 (3)	−0.015 (3)
C13	0.077 (3)	0.085 (3)	0.089 (3)	−0.018 (3)	0.027 (2)	−0.006 (3)
C14	0.157 (4)	0.110 (4)	0.106 (4)	0.039 (3)	0.073 (3)	−0.011 (3)
C15	0.076 (3)	0.048 (2)	0.058 (3)	0.002 (2)	0.020 (2)	−0.004 (2)
C16	0.061 (2)	0.044 (2)	0.053 (2)	−0.0026 (18)	0.0124 (19)	−0.0024 (19)
C17	0.058 (2)	0.053 (2)	0.043 (2)	−0.0057 (19)	0.0038 (18)	0.0003 (19)
C18	0.059 (2)	0.058 (2)	0.045 (2)	0.005 (2)	0.0057 (19)	0.001 (2)
C19	0.064 (3)	0.078 (3)	0.058 (3)	0.003 (2)	0.007 (2)	0.008 (2)
C20	0.088 (3)	0.079 (3)	0.052 (3)	−0.009 (2)	−0.002 (2)	−0.007 (2)
C21	0.088 (3)	0.068 (3)	0.055 (3)	−0.001 (2)	0.002 (2)	−0.015 (2)
C22	0.075 (3)	0.090 (3)	0.086 (3)	0.034 (2)	0.013 (2)	−0.002 (3)

### Geometric parameters (Å, °)

**Table:**

N1—C7	1.272 (4)	C8—C13	1.383 (4)
N1—C1	1.419 (4)	C9—C10	1.391 (4)
N2—C15	1.270 (4)	C10—C11	1.360 (5)
N2—C2	1.389 (4)	C11—C12	1.368 (5)
O1—C9	1.330 (4)	C11—H11	0.930
O1—H1	0.820	C12—C13	1.357 (5)
O2—C10	1.342 (4)	C12—H12	0.930
O2—C14	1.409 (4)	C13—H13	0.930
O3—C17	1.339 (3)	C14—H14A	0.960
O3—H3	0.820	C14—H14B	0.960
O4—C18	1.360 (4)	C14—H14C	0.960
O4—C22	1.413 (3)	C15—C16	1.417 (4)
C1—C6	1.370 (4)	C15—H15	0.930
C1—C2	1.381 (4)	C16—C21	1.385 (4)
C2—C3	1.381 (4)	C16—C17	1.395 (4)
C3—C4	1.358 (4)	C17—C18	1.377 (4)
C3—H3A	0.930	C18—C19	1.351 (4)
C4—C5	1.358 (5)	C19—C20	1.385 (4)
C4—H4	0.930	C19—H19	0.930
C5—C6	1.365 (5)	C20—C21	1.354 (4)
C5—H5	0.930	C20—H20	0.930
C6—H6	0.930	C21—H21	0.930
C7—C8	1.439 (4)	C22—H22A	0.960
C7—H7	0.930	C22—H22B	0.960
C8—C9	1.370 (4)	C22—H22C	0.960
			
C7—N1—C1	118.1 (3)	C13—C12—C11	120.3 (4)
C15—N2—C2	123.4 (3)	C13—C12—H12	119.8
C9—O1—H1	109.5	C11—C12—H12	119.8
C10—O2—C14	117.7 (3)	C12—C13—C8	120.2 (4)
C17—O3—H3	109.5	C12—C13—H13	119.9
C18—O4—C22	117.7 (3)	C8—C13—H13	119.9
C6—C1—C2	119.9 (4)	O2—C14—H14A	109.5
C6—C1—N1	121.9 (4)	O2—C14—H14B	109.5
C2—C1—N1	118.1 (3)	H14A—C14—H14B	109.5
C1—C2—C3	119.1 (4)	O2—C14—H14C	109.5
C1—C2—N2	116.6 (3)	H14A—C14—H14C	109.5
C3—C2—N2	124.3 (4)	H14B—C14—H14C	109.5
C4—C3—C2	119.9 (4)	N2—C15—C16	121.6 (3)
C4—C3—H3A	120.1	N2—C15—H15	119.2
C2—C3—H3A	120.1	C16—C15—H15	119.2
C5—C4—C3	121.1 (4)	C21—C16—C17	118.3 (3)
C5—C4—H4	119.4	C21—C16—C15	120.8 (4)
C3—C4—H4	119.4	C17—C16—C15	120.9 (3)
C4—C5—C6	119.7 (4)	O3—C17—C18	118.1 (3)
C4—C5—H5	120.2	O3—C17—C16	121.6 (3)
C6—C5—H5	120.2	C18—C17—C16	120.4 (3)
C5—C6—C1	120.4 (4)	C19—C18—O4	125.7 (3)
C5—C6—H6	119.8	C19—C18—C17	119.9 (4)
C1—C6—H6	119.8	O4—C18—C17	114.4 (3)
N1—C7—C8	121.8 (4)	C18—C19—C20	120.5 (4)
N1—C7—H7	119.1	C18—C19—H19	119.8
C8—C7—H7	119.1	C20—C19—H19	119.8
C9—C8—C13	119.5 (4)	C21—C20—C19	120.1 (3)
C9—C8—C7	122.0 (4)	C21—C20—H20	120.0
C13—C8—C7	118.5 (4)	C19—C20—H20	120.0
O1—C9—C8	122.5 (4)	C20—C21—C16	120.8 (4)
O1—C9—C10	117.6 (4)	C20—C21—H21	119.6
C8—C9—C10	119.9 (4)	C16—C21—H21	119.6
O2—C10—C11	125.5 (4)	O4—C22—H22A	109.5
O2—C10—C9	115.1 (4)	O4—C22—H22B	109.5
C11—C10—C9	119.4 (4)	H22A—C22—H22B	109.5
C10—C11—C12	120.6 (4)	O4—C22—H22C	109.5
C10—C11—H11	119.7	H22A—C22—H22C	109.5
C12—C11—H11	119.7	H22B—C22—H22C	109.5

### Hydrogen-bond geometry (Å, °)

**Table:**

*D*—H···*A*	*D*—H	H···*A*	*D*···*A*	*D*—H···*A*
O1—H1···N1	0.82	1.88	2.605 (4)	146
O3—H3···N2	0.82	1.82	2.542 (3)	146
